# Health-related quality of life (HRQoL) in a population at risk of type 2 diabetes: a cross-sectional study in two Latin American cities

**DOI:** 10.1186/s12955-021-01894-7

**Published:** 2021-12-20

**Authors:** Luis A. Anillo Arrieta, Tania Acosta Vergara, Rafael Tuesca, Sandra Rodríguez Acosta, Karen C. Flórez Lozano, Pablo Aschner, Rafael Gabriel, Sandra De La Rosa, Julieth P. Nieto Castillo, Noël C. Barengo

**Affiliations:** 1 Department of Mathematics and Statistics, Division of Basic Sciences, Universidad del Norte, Barranquilla, Colombia; 2 Department of Public Health, Division of Health Sciences, Universidad del Norte, Barranquilla, Colombia; 3 Department of Interdisciplinary Research, University Center CIFE, Cuernavacas-Morelos, Mexico; 4 Department of Economics, Division of Humanities and Sciences Division Social, Universidad del Norte, Barranquilla, Colombia; 5 Asociación Colombiana de Diabetes, Bogotá, Colombia; 6 Javeriana University, Bogotá, Colombia; 7 San Ignacio University Hospital, Bogotá, Colombia; 8 Department of International Health, National School of Public Health, Instituto de Salud Carlos III, Madrid, Spain; 9 World Community for Prevention of Diabetes (WCPD) Foundation, Madrid, Spain; 10 Department of Translational Medicine, Herbert Wertheim College of Medicine, Florida International University, Miami, FL USA; 11 Department of Public Health, Faculty of Medicine, University of Helsinki, Helsinki, Finland; 12 Department of Health Policy and Management, Robert Stempel College of Public Health and Social Work, Florida International University, Miami, FL USA

**Keywords:** Health related quality of life, Hyperglycemia, Type 2 diabetes, Risk factors

## Abstract

**Purpose:**

The purpose of this study was to describe the health-related quality of life (HRQoL) characteristics in a population at risk of developing type 2 diabetes in Barranquilla and Bogotá, Colombia.

**Methods:**

A cross-sectional study with 1135 participants older than 30 years-of-age recruited in Bogotá D.C., and Barranquilla by cluster sampling in 2018 to 2019. The Finnish Diabetes Risk Score (FINDRISC) was used to detect participants at risk of developing type 2 diabetes (T2D). HRQoL was assessed using the EQ-5D-3L questionnaire. Unadjusted and adjusted logistic regression models were used to calculate odds ratios (OR) and their corresponding 95% confidence intervals CI).

**Results:**

Moderate or extreme problems appeared more frequently in the dimensions of Pain/Discomfort (60.8%) and Anxiety/Depression (30.8%). The mean score of the EQ-VAS was 74.3 (± 17.3), significantly larger in the state of complete health (11111) compared with those with problems in more than one of the quality-of-life dimensions. Being female and living in Bogota D.C., were associated with greater odds of reporting problems in the Pain (OR 1.6; 95% CI 1.2–2.2) and Discomfort dimensions (OR 1.6; 95% CI 1.2–2.0) respectively and Anxiety/Depression (OR 1.9; 95% CI 1.3–2.7), (OR 9.1; 95% CI 6.6–12.4), respectively.

**Conclusions:**

As living place and sex were associated with dimensions of Pain/Discomfort and Anxiety/Depression in the HRQoL in people at risk of T2D, greater attention should be paid to these determinants of HRQoL to design and reorient strategies with a territorial and gender perspective to achieve better health outcomes.

**Plain English summary:**

Diabetes is one of the four non-communicable diseases with increasing prevalence in the world, which has made it a serious public health problem. In Colombia, in 2019 diabetes affected 8.4% of the Colombian adult population and more than one million Colombian adults of this age group have hidden or undetected diabetes. This disease is not only characterized by increased premature mortality, loss of productivity, and economic impact, but it also involves a deterioration in the quality of life of people with diabetes with their respective families. However, very Little is known about health-related quality of life (HRQoL) in a population at risk or with prediabetes. This study has evaluated the quality of life in patients at risk of diabetes and their behavior with some variables as sociodemographic, lifestyle, history, and established their difference in two territories of the Colombian Caribbean. The results of this study indicate that the HRQoL of people at risk of type 2 diabetes is affected by factors such as gender, city, dysglycemia, medication for hypertension and education level. Therefore, greater attention should be paid to these determinants of HRQL to design and implement strategies that reduce this risk of developing type 2 diabetes, prevent prediabetes and improve the quality of life in prediabetic or diabetic patients.

## Introduction

It is estimated that 463 million adults around the world have diabetes and 374 million have prediabetes. It is expected that by 2045 the numbers of adults with diabetes and prediabetes will be 700 and 548 million, respectively. In Colombia, the prevalence of diabetes in the adult population is 8.4% and it is estimated that more than one million have undiagnosed diabetes [1, 2]. Randomized clinical trials have shown the potential to prevent or delay the development of type 2 diabetes (T2D) in people at risk of developing the disease through lifestyle changes that included changes in eating habits and physical activity [3–5]. However, the first step in preventing diabetes is identifying people at risk while they are still in a normoglycemic state [6].

The measurement of blood glucose levels is the method commonly used for the identification of subjects at risk of diabetes, however, it is an invasive, time-consuming and expensive process, therefore, it is currently available of the diabetes risk score (FINDRISC) which allows identifying people with future risk of T2D [6, 7]. The FINDRISC scoring system was developed in Finland and is an instrument that has been adapted and validated in many countries including ours. In Colombia, a FINDRISC score ≥ 12 is considered diabetes risk [8, 9].

Several studies have established that T2D significantly reduces the health-related quality of life (HRQoL) [10–14]. However, there is little and controversial literature on HRQoL in patients at risk for T2D [15–19]. Previous studies describing HRQoL in persons at risk for diabetes only included participants with impaired glucose tolerance or prediabetes [15–19]. However, these studies did not include people at future risk of developing T2D that may still have normal glucose levels [6].

HRQoL is the measure most frequently used to evaluate the impact of chronic illness or its treatment. The use of generic questionnaires such as the EQ-5D [20–24], SF-36 [25], and Health Utilities Index [26] have become the most common methods to establish the effects in terms of HRQoL. In the recent scientific literature, the EQ-5D tends to be the most widely used instrument in cost-utility analyses [27], not only because it is a short and cognitively simple questionnaire to complete but also because it is the instrument preferred by evaluation agencies of health technologies to establish measures of HRQoL in adults [28, 29]. To our knowledge, there is no information available on HRQL in a population at risk of T2D through FINDRISC in Latin America. The objective of this study was to describe the characteristics of health-related quality of life (HRQoL) in a population at risk of developing type 2 diabetes in Barranquilla and Bogotá, Colombia.

## Materials and methods

### Study design and population

This cross-sectional study used baseline information collected within the PREDICOL project (Evaluation of a community health program for the prevention of type 2 diabetes and other cardio-metabolic risk factors in adults) developed in neighborhoods of two Colombian cities. In Bogotá, the capital city of Colombia with the largest number of inhabitants (7,743,955 inhabitants approximately), located in the central region of the country at 2630 m above sea level with a climate that ranges between 5 and 19 °C, the project It was developed in the Palermo Sur neighborhood located in the southeast of the capital, has 50,000 inhabitants and has approximately 23.5 ha of land, mostly mountainous terrain and Barranquilla, the fourth most populated city in the country with 1,274,250 inhabitants (2.5% of the total population of Colombia) located in the Colombian Caribbean region above sea level with an average temperature of 29 °C, was developed in the El Pueblo neighborhood, located in the south west of the city with 240,000 inhabitants with flat land [30].

The study participants were recruited in Bogota and Barranquilla by cluster sampling [30]. During January 2018 to December 2019 approximately 1350 households of lower socio-economic strata were visited. All persons older than 30 years-of-age in each household were asked to fill in the Finnish Diabetes Risk Score (FINDRISC). In total, the FINDRISC was applied to 3738 people over 30 years-of-age. Respondents with a FINDRISC ≥ 12 points were invited to the health care centers of each location for an oral glucose tolerance test (OGTT), managing to establish the glycemic status of 1166 participants (55% of the 2118 at risk of T2D). This study excluded people with known type 2 diabetes, cognitive disabilities, or pregnant women. Further, the records of the participants with missing data (31 records with some missing data—2.6%) in the variables used in this analysis and in the FINDRISC, HRQoL and physical activity questionnaires were excluded. The final sample included 1135 participants with completed questionnaires and who met previously defined criteria. This study was registered at ClinicalTrials.gov under the identification number NCT03049839. The methodology of this study has been published previously in a recent publication [31].

### Non-invasive measurements

Before the blood glucose measurements, the participants' HRQoL was assessed using the Spanish version of the generic EQ-5D-3L questionnaire from the EuroQol group. This questionnaire consists of two parts, the EQ-5D descriptive component and the visual analog scale (EQ-VAS) [20, 21]. The descriptive component contains five dimensions: Mobility (MO), Self-Care (SC), Usual Activities (UA), Pain/Discomfort (P/D), and Anxiety/Depression (A/D). Each dimension consists of three levels: No problems (1), moderate problems (2), and extreme or severe problems (3), which are self-reported by the participant according to their health state. Combination of the digits of all the dimensions generates a 5-digit number that describes the health state of the participant, giving 243 possible states [20, 22]. For example, state 11111 indicates no problem in any of the five dimensions, whereas state 11122 indicates the following: No problems walking (1), no problems with personal care (1), no problems with daily activities (2), moderate pain or discomfort (2), and moderate anxiety or depression (1). Many countries have developed a set of values or rates for all possible health states of the EQ-5D-3L using many preference-based techniques, however, Colombia does not yet have a locally appropriate set of values as suggested by the EuroQol group [22–24].

The second part of the EQ-5D is the EQ-VAS which records self-rated health on a millimeter scale ranging from 0 (worst imaginable health) to 100 (best imaginable health). The participant had to mark the point on a vertical line that best reflects the assessment of their overall health state [20, 22]. In this study, due to the absence of a locally appropriate set of values, the EQ-5D score was not calculated, therefore only the frequencies of the EQ-5D-3L health states were described and the HRQoL of participants was evaluated using the EQ-VAS.

The risk of T2D was assessed using the FINDRISC, which consists of eight items: age, body mass index (BMI), waist circumference, self-report of physical activity of at least 30 min per day, daily consumption of fruits or vegetables, history of high blood glucose, and family history of T2D [6, 7]. The total risk score is a simple sum of the individual weights, with a minimum score of 0 and maximum of 26. This questionnaire has been validated in various countries including Colombia, and a score of ≥ 12 has been used to define at risk of future T2D [8, 9, 32, 33]. For determining the physical activity (PA) level, the short version of the International Physical Activity Questionnaire (IPAQ-SV) was used, comprising seven questions addressing the frequency, intensity, and duration of physical activity during the last 7 days [34–37]. Data were also collected through a socio-demographic questionnaire that included information on the marital status, occupation and educational level of the participants.

### Invasive measurements

Participants scoring ≥ 12 on the FINDRISC were invited to an oral glucose tolerance test according to recommendations of the World Health Organization (WHO) [38, 39]. The test started after 12 h fasting, and the fasting and 2-h blood samples were obtained after oral ingestion of water solution with 75 g anhydrous glucose. The glucose tolerance status was classified according to the WHO criteria [38]. Individuals who had fasting plasma glucose (FPG) level ≥ 126 mg/dl or 2 h plasma glucose (2hPG) ≥ 200 mg/dl were classified as having Type 2 diabetes mellitus. Those with 2hPG ≥ 140 mg/dl but < 200 mg/dl, and FPG < 100 mg/dl were classified as having isolated IGT. Isolated IFG was defined as FPG ≥ 110 but < 126 mg/dl, and 2hPG < 140 mg/dl. People with 2hPG ≥ 140 mg/dl but < 200 mg/dl, and FPG ≥ 110 but < 126 mg/dl were defined as combined IGT and IFG. Abnormal glucose tolerance was defined as T2D, IGT or IFG. [38, 40].

### Ethical aspects

This study followed the rules of good clinical practice and guidelines of the Declaration of Helsinki. It was approved by the ethics committee of the Universidad del Norte (report 141 of 28 April 2016). Each participant signed the informed consent before participating and could withdraw from the study at any time they preferred.

### Statistical analysis

Categorical variables and frequency distributions by health state were expressed in absolute numbers and percentages with 95% confidence intervals (95% CI). Quantitative variables were summarized by means and standard deviations and the evaluation expressed on a visual analog scale (EQ-VAS), with the most frequent health states using density graphs. Normality of the variables was verified through Kolmogorov–Smirnov tests. The nonparametric U tests of Mann–Whitney and Kruskal–Wallis were used to evaluate statistical differences between subgroups, and the chi-square test (X^2^) for differences in proportions (homogeneity test). To establish factors associated with presenting problems in the quality-of-life dimensions of the EQ-5D-3L descriptive system, several binary logistic regression models were tested. In the logistic regression model, the dependent variable was each domain of the quality-of-life questionnaire, previously dichotomized into *no problems* (level 1) and *with some problem* (levels 2 and 3). To summarize the data, only independent variables that showed a significant association with the quality-of-life dimensions were reported. The results are presented according to sex, city, and age group. *p*-values < 0.05 were considered statistically significant. Processing and analysis of the data were carried out using statistical software SPSS version 25 (SPSS; Chicago, IL, USA), with the density graphs constructed using R version 4.0.0.

## Results

Table 1 presents the baseline characteristics of the study participants. The final study population included 1135 participants from two cities in Colombia (Barranquilla, n = 587; Bogotá D.C., n = 548). Of these, 54.6% were older than 54 years, and the majority were women (76.4%). The percentage of participants with prediabetes was 16% (n = 182), with more frequent occurrence in participants from Barranquilla. The proportion of hidden diabetes was 9%, with statistical equality between cities. There was 87.7% of the population presenting overweight or obese. Most participants had a low educational level (no education or not finishing high school, 67.2%). The proportion of participants with little physical activity was larger in Barranquilla (91.7%). The percentages of current smokers and those who reported receiving treatment for hypertension were 7.8% and 38.5%, respectively.

**Table 1:** Baseline characteristics of population at risk of T2D in Barranquilla and Bogotá, Colombia in 2018

Variable	Total 1135 (100)	City		
		Barranquilla 587 (51.7)	Bogota D.C 548 (48.3)	*p*-value^a^
*Gender*				
Male	268 (23.6)	143 (24.4)	125 (22.8)	0.539
Female	867 (76.4)	444 (75.6)	423 (77.2)	
*Age groups (years)*				
30–44	226 (19.9)	140 (23.9)	86 (15.7)	< 0.001
45–54	289 (25.5)	168 (28.6)	121 (22.1)	
55–64	338 (29.8)	164 (27.9)	174 (31.8)	
> 64	282 (24.8)	115 (19.6)	167 (30.5)	
*Education level*				
No schooling	287 (25.3)	97 (16.5)	190 (34.7)	< 0.001
Elementary school	475 (41.9)	247 (42.1)	228 (41.7)	
Junior high school	247 (21.8)	161 (27.4)	86 (15.7)	
Higher	125 (11.0)	82 (14.0)	43 (7.9)	
*Physical activity*				
Low	854 (75.2)	538 (91.7)	316 (57.7)	< 0.001
Moderate	186 (16.4)	30 (5.1)	156 (28.5)	
High	95 (8.4)	19 (3.2)	76 (13.9)	
*BMI (kg/m*^*2*^*)*				
Normal weight	140 (12.3)	67 (11.4)	73 (13.3)	0.006
Overweight	467 (41.1)	220 (37.5)	247 (45.1)	
Obesity	528 (46.5)	300 (51.1)	228 (41.6)	
*Glucose classification*				
IFG, IGT or IFG + IGT	182 (16.0)	110 (18.7)	72 (13.1)	0.002
T2D	102 (9.0)	63 (10.7)	39 (7.1)	
*Hypertension treatment*				
Yes	437 (38.5)	226 (38.5)	211 (38.5)	0.999
*Current smokers*				
Yes	88 (7.8)	24 (4.1)	64 (11.8)	< 0.001

^a^Homogeneity test (X^2^); n (%). BMI, body mass index; IFG, impaired fasting glucose; IGT, impaired glucose tolerance; T2D, type 2 diabetes

The percentages of study participants expressing problems in each dimension of the EQ-5D-3L description system are shown in Table 2. Altogether, 21.7% reported mobility problems, 2.0% self-care, 15.1% daily activities, 60.8% pain/discomfort, and 30.8% anxiety/depression. The most frequent changes were the presence of moderate pain or discomfort (53.9%; 95% CI 51.0–56.8), followed by moderately anxiety or depression (26.5%; 95% CI 24.0–29.2) and some problems walking. Reports of moderate pain or discomfort were statistically more numerous among women, without differentiation between cities. The proportion of participants who reported being moderately anxious or depressed was significantly higher among women and residents of Bogotá D.C. The percentage of participants who had trouble walking was larger among residents of Bogotá.

**Table 2:** Distribution of frequency response for each dimension of EQ-5D-3L

Dimensions	Total		Gender				City			
			Female		Male		Barranquilla		Bogota D.C	
	n (%)	95% CI	n (%)	95% CI	n (%)	95% CI	n (%)	95% CI	n (%)	95% CI
*Mobility*										
I have no problems walking about	889 (78.3)	75.8–80.6	674 (77.7)	74.9–80.4	215 (80.2)	75.0–84.8	538 (91.7)	89.1–93.6	351 (64.1)	60.0–68.0*
I have some problems walking about	244 (21.5)	19.2–24.0	192 (22.2)	19.5–25.0	52 (19.4)	14.8–24.7	49 (8.3)	6.4–10.9	195 (35.6)	31.7–39.7*
I am confined to bed	2 (0.2)	0.05–0.60	1 (0.1)	0.02–0.7	1 (0.4)	0.01–2.1	0 (0)	–	2 (0.4)	0.1–1.3
*Self-care*										
I have no problems with self-care	1112 (98.0)	97.0–98.7	847 (97.7)	96.5–98.5	265 (98.9)	96.8–99.8	584 (99.5)	98.5–99.8	528 (96.3)	94.4–97.6*
I have some problems washing or dressing myself	21 (1.8)	1.2–2.8	19 (2.2)	1.4–3.4	2 (0.7)	0.09–2.7	3 (0.5)	0.2–1.5	18 (3.3)	2.1–5.1*
I am unable to wash or dress myself	2 (0.2)	0.05–0.60	1 (0.1)	0.02–0.7	1 (0.4)	0.01–2.1	0 (0)	–	2 (0.4)	0.1–1.3
*Usual activities*										
I have no problems with performing my usual activities	964 (84.9)	82.7–86.9	729 (84.1)	82.0–86.4	235 (87.7)	83.1–91.4	566 (96.4)	94.6–97.7	398 (72.6)	68.8–76.2*
I have some problems with performing my usual activities	169 (14.9)	12.9–17.1	137 (15.8)	13.5–18.4	32 (11.9)	8.3–16.4	21 (3.6)	2.4–5.4	148 (27.0)	23.5–30.9*
I am unable to perform my usual activities	2 (0.2)	0.05–0.64	1 (0.1)	0.02–0.65	1 (0.4)	0.01–2.1	0 (0)	–	2 (0.4)	0.1–1.3
*Pain/discomfort*										
I have no pain or discomfort	445 (39.2)	36.4–42.1	315 (36.3)	33.2–39.6	130 (48.5)	42.4–54.7 *	260 (44.3)	40.3–48.3	185 (33.8)	29.9–37.8*
I have moderate pain or discomfort	612 (53.9)	51.0–56.8	487 (56.2)	52.9–59.4	125 (46.6)	40.6–52.8 *	323 (55.0)	51.0–59.0	289 (52.7)	48.6–56.9
I have extreme pain or discomfort	78 (6.9)	5.5–8.5	65 (7.5)	5.9–9.4	13 (4.9)	2.6–8.2	5 (0.7)	0.3–1.7	74 (13.5)	10.9–16.6*
*Anxiety/depression*										
I am not anxious or depressed	785 (69.2)	66.4–71.8	577 (66.6)	63.3–69.6	208 (77.6)	72.1–82.5 *	523 (89.1)	86.3–91.4	262 (47.8)	43.7–52.0*
I am moderately anxious or depressed	301 (26.5)	24.0–29.2	248 (28.6)	25.7–31.7	53 (19.8)	15.2–25.1 *	59 (10.1)	7.9–12.8	242 (44.2)	40.1–48.3*
I am extremely anxious or depressed	49 (4.3)	3.3–5.7	42 (4.8)	3.6–6.5	7 (2.6)	1.1–5.3	5 (0.9)	0.4–2.0	44 (8.0)	6.0–10.6*

*Difference is statistically significant at the 0.05 level

Participants reported their health in 45 health states (SQoL). However, of the 243 possible, 81.1% reported their current health in only seven SQoL. Table 3 shows the percentages of the most reported health states (SQoL) in this study. The percentages are also shown by sex and city. Most of the participants (69.4%) reported some problem in the dimensions of the EQ-5D-3L questionnaire. The most reported health status was *complete health* (SQoL: 11111, 30.6%; 95% CI 28.0–33.3), The percentage of participants free of health complains was statistically significantly higher in men than in women (39% vs 28%), and those living in Barranquilla compared with participants in Bogota (40% vs 20%). The second most reported health status was moderate pain or discomfort, with no problems in the other four dimensions (SQoL: 11121, 27%; 95% CI 24.5–29.6), with similarity by sex. However, a higher prevalence of moderate pain/discomfort was found in participants in Barranquilla compared with those in Bogotá (*p*-value < 0.05). Moderate pain / discomfort and moderate anxiety / depression with no problems in the other dimensions (SQoL: 11122) was presented 8% of the study participants. The SQoL: 11122 were more prevalent in women and those living in Bogotá compared to their counterparts. The percentage of those who presented some walking problems and moderate pain or discomfort without presenting problems in the other dimensions (SQoL: 21121) did not differ significantly by sex (3.8% vs 3.4%) and by city (3.9% vs 3.5%). Approximately 10% (n = 114) presented states with more severe alterations (three in any of the dimensions), a very large proportion compared to that found in the general Colombian population [41].

**Table 3:** Assessment of current health state for population at risk of T2D, by gender and city

SQoL	Total *n* = 1135		Gender				City			
			Women *n* = 867		Men *n* = 268		Barranquilla *n* = 587		Bogota D.C. *n* = 548	
	n (%)	95% CI	n (%)	95% CI	n (%)	95% CI	n (%)	95% CI	n (%)	95% CI
11111	347 (30.6)	28.0–33.3	243 (28.0)	25.1–31.1	104 (38.8)	32.9–44.9*	237 (40.4)	36.5–44.4	110 (20.1)	16.9–23.6*
11121	306 (27.0)	24.5–29.6	237 (27.3)	24.5–30.4	69 (25.8)	20.6–31.4	240 (40.9)	37.0–44.9	66 (12.0)	9.6–15.0*
11122	91 (8.0)	6.6–9.7	79 (9.1)	7.4–11.2	12 (4.5)	2.3–7.7*	31 (5.3)	3.8–7.4	60 (11.0)	8.6–13.8*
11112	57 (5.0)	3.9–6.5	44 (5.1)	3.8–6.7	13 (4.9)	2.6–8.2	12 (2.0)	1.2–3.5	45 (8.2)	6.2–10.8*
21222	45 (4.0)	3.0–5.3	35 (4.0)	2.9–5.6	10 (3.7)	1.8–6.8	3 (0.5)	0.2–1.5	42 (7.7)	5.7–10.2*
21121	42 (3.7)	2.8–5.0	33 (3.8)	2.7–5.3	9 (3.4)	1.6–6.3	23 (3.9)	2.6–5.8	19 (3.5)	2.2–5.4
21122	32 (2.8)	2.0–4.0	26 (3.0)	2.1–4.4	6 (2.2)	0.8–4.8	7 (1.2)	0.6–2.4	25 (4.6)	3.1–6.7 *
38 other states	215 (18.9)	16.8–21.3	170 (19.6)	17.1–22.4	45 (16.8)	12.5–21.8	34 (5.8)	4.2–8.0	181 (33.0)	29.2–37.1 *

*Statistically significant difference *p*-value < 0.05 (homogeneity test). 11111, without problems in all five dimensions; 11121, moderate pain/discomfort with no problems in the other four dimensions; 11122, moderate pain/discomfort and moderate anxiety/depression without problems in the other three dimensions; 11112, moderate anxiety/depression without problems in the other four dimensions; 21222, some trouble walking, no self-care problems, some trouble performing usual activities, moderate pain/discomfort, and moderate anxiety/depression; 21121, some walking problems and moderate pain/discomfort with no problems in the other three dimensions; 21122, some walking problems, moderate pain/discomfort and moderate anxiety/depression without problems in the other two dimensions

**Fig. 1:**
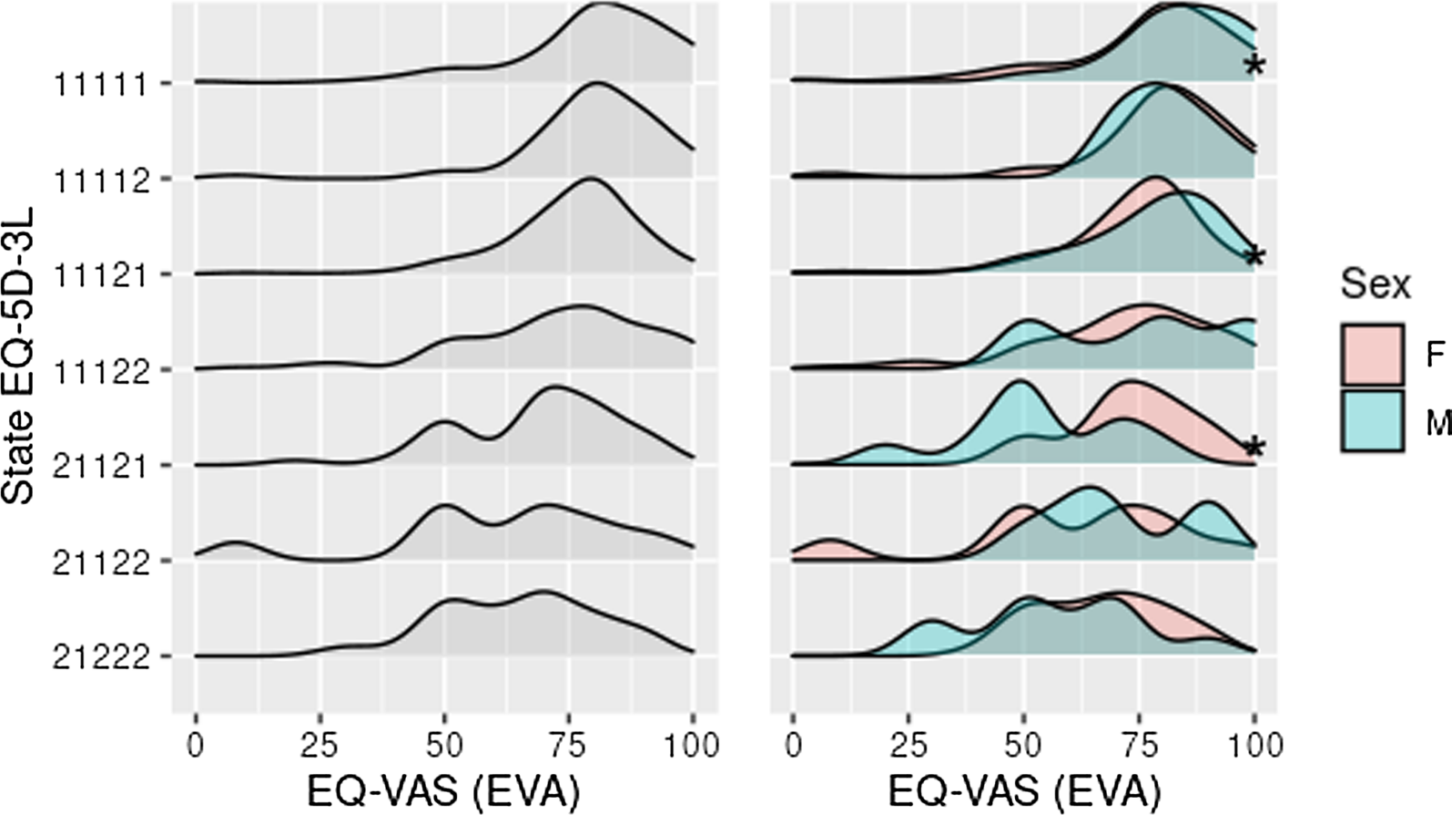
Distribution of EQ-VAS score for most frequent health states in the population at risk of suffering T2D in Barranquilla and Bogotá, Colombia. ^a^HRQoL distribution in the general population and by sex. *Statistically significant difference; T2D, type 2 diabetes; 11111, without problems in all five dimensions; 11121, moderate pain/discomfort with no problems in the other four dimensions; 11122, moderate pain/discomfort and moderate anxiety/depression without problems in the other three dimensions; 11112, moderate anxiety/depression without problems in the other four dimensions; 21222, some trouble walking, no self-care problems, some trouble performing usual activities, moderate pain/discomfort, and moderate anxiety/depression; 21121, some walking problems and moderate pain/discomfort with no problems in the other three dimensions; 21122, some walking problems, moderate pain/discomfort and moderate anxiety/depression, without problems in the other two dimensions

**Fig. 2:**
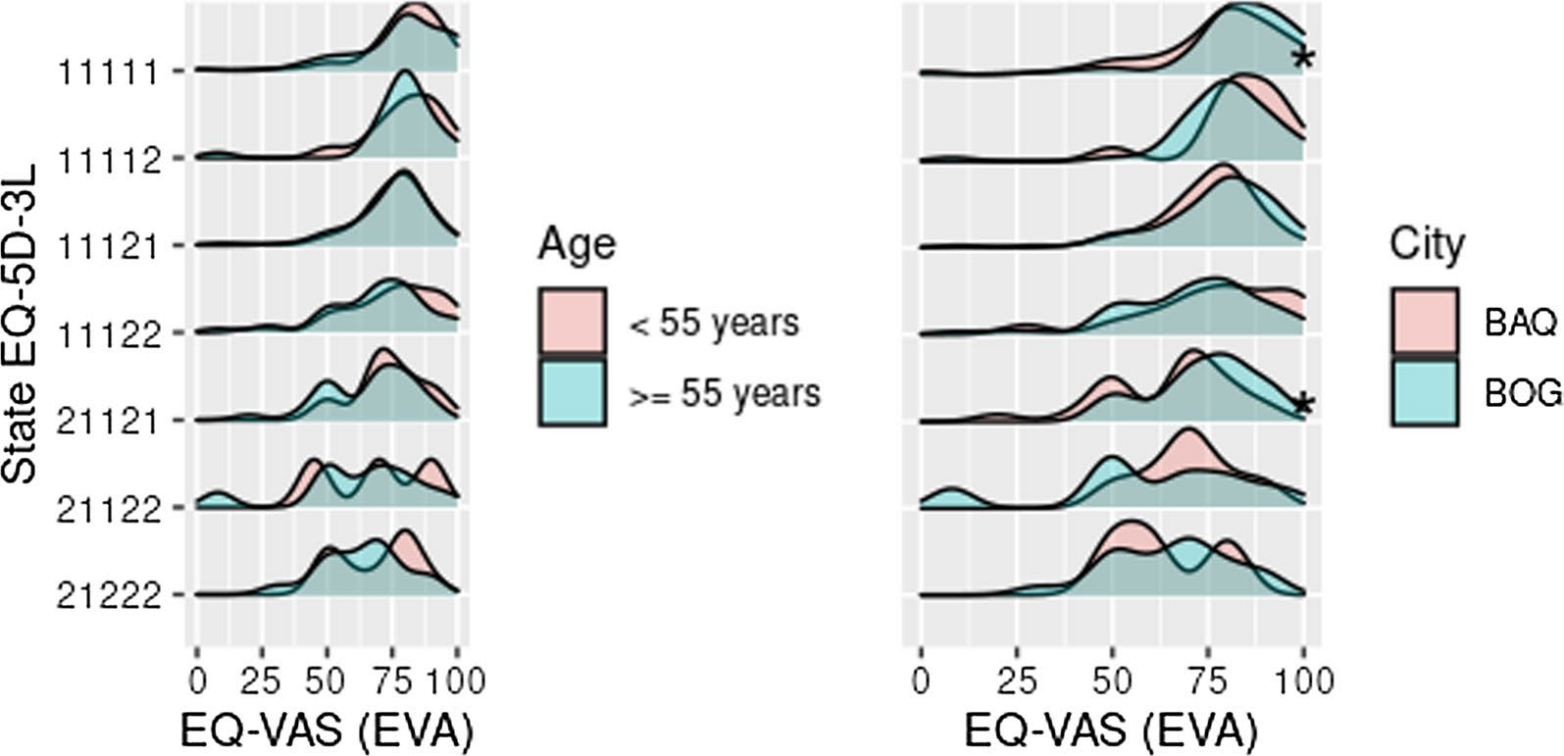
Distribution of EQ-VAS score for the most frequent health states in the population at risk of T2D, according to city and age group. ^a^HRQoL distribution according to age group and city; *Statistically significant difference; T2D, type 2 diabetes; BAQ, Barranquilla; BOG, Bogotá; 11111, without problems in all five dimensions; 11121, moderate pain/discomfort with no problems in the other four dimensions; 11122, moderate pain/discomfort and moderate anxiety/depression without problems in the other three dimensions; 11112, moderate anxiety/depression without problems in the other four dimensions; 21222, some trouble walking, no self-care problems, some trouble performing usual activities, moderate pain/discomfort, and moderate anxiety/depression; 21121, some walking problems and moderate pain/discomfort with no problems in the other three dimensions; 21122, some walking problems, moderate pain/discomfort and moderate anxiety/depression, with no problems in the other two dimensions

The scores obtained on the EQ-VAS for the seven most reported SQoLs show that the health state complete and states presenting moderate problems in one of the dimensions of quality of life had higher scores, in contrast to states presenting problems in more than one dimension (left part of Fig. 1). The average score for the complete health state was 79.3 ± 17.1 (SQoL: 11111), and the corresponding value for women was smaller than for men (*p*-value < 0.05). Similarly, women reported statistically significantly smaller values in SQoL 11121 compared to men. For SQoLs 11122, 21121, 21122 and 21222, no significant differences were detected, except in SQoL 21121 (some walking problems and moderate pain or discomfort, without problems in the other three dimensions), for which the average score was 53.3 ± 18 among men—statistically significantly smaller than the score for women (73.2 ± 12.9) (see right part of Fig. 1). Regarding living place, it was observed that the participants from Barranquilla (SQoL 11111) had lower values for their health state compared with those from Bogotá D.C., 78 ± 16.7 and 82.1 ± 17.6, respectively. For the other six SQoLs, no statistically significant differences were found in values determined by the EQ-VAS. The scores of people older than 55 were comparable to those of participants of younger age-groups even when they reported no health problems (Fig. 2).

Table 4 reveals the results of the logistic regression models for each dimension of the EQ-5D-3L, in which the city (OR 5.9; 95% CI 4.2–8.4), age (OR 2.5; 95% CI 1.8–3.5) and hypertension treatment (OR 1.6; 95% CI 1.2–2.2) were statistically significant predictors of MO. In addition, living place (OR 8.2; 95% CI 2.4–27.9) and impaired glucose tolerance (OR 2.4; 95% CI 1.04–5.8) were associated with SC. Moreover, living place, age, receipt of treatment for hypertension, and education were associated with UA. The problems in the dimension of P/D was greater among participants from Bogotá (OR = 1.6; 95% CI 1.2–2.0) and women (OR 1.6; 95% CI 1.2–2.2). The odds of A/D was nine times higher in participants from Bogotá (OR 9.1, 95% CI 6.6–12.4) compared with those from Barranquilla and almost twice as in women (OR 1.9, 95% CI 1.3–2.7) than men.

**Table 4:** Factors associated with presenting problems in the of quality of life dimensions for the population at risk of T2D in Barranquilla and Bogota

Dependent variable	Characteristic	B	SE	*p*-value	OR (95% CI)
Mobility	*City*				
	Barranquilla (Ref.)				
	Bogota D.C	1.78	0.18	< 0.001	5.9 (4.2–8.4)
	*Age group*				
	< 55 years (Ref.)				
	≥ 55 years	0.91	0.18	< 0.001	2.5 (1.8–3.5)
	*Hypertension treatment*				
	No (Ref.)				
	Yes	0.48	0.16	0.003	1.6 (1.2–2.2)
Self-care	*City*				
	Barranquilla (Ref.)				
	Bogota D.C	2.1	0.63	0.001	8.2 (2.4–27.9)
	*Glucose classification*				
	Normal (Ref.)				
	Impaired glucose tolerance	0.89	0.44	0.041	2.4 (1.04–5.8)
Usual activities	*City*				
	Barranquilla (Ref.)				
	Bogota D.C	2.2	0.25	< 0.001	9.3 (5.7–15.2)
	*Age group*				
	< 55 years (Ref.)				
	≥ 55 years	0.78	0.22	< 0.001	2.2 (1.4–3.3)
	*Hypertension treatment*				
	No (Ref.)				
	Yes	0.41	0.19	0.028	1.5 (1.05–2.2)
	*Education level*				
	Superior (Ref.)				
	No schooling	− 0.373	0.34	0.265	0.69 (0.36–1.3)
	Elementary school	− 0.671	0.32	0.039	0.51 (0.27–0.97)
	Junior high school	− 0.979	0.39	0.011	0.38 (0.18–0.8)
Pain/discomfort	*City*				
	Barranquilla (Ref.)				
	Bogota D.C	0.442	0.12	< 0.001	1.6 (1.2–2.0)
	*Sex*				
	Male (Ref.)				
	Female	0.50	0.14	< 0.001	1.6 (1.2–2.2)
Anxiety/depression	*City*				
	Barranquilla (Ref.)				
	Bogota D.C	2.2	0.16	< 0.001	9.1 (6.6–12.4)
	*Sex*				
	Male (Ref.)				
	Female	0.63	0.18	< 0.001	1.9 (1.3–2.7)

^a^OR, Odds Ratio; Ref, reference group

## Discussion

Our data indicate that gender, age, city, impaired glucose tolerance, treatment for hypertension, and educational level was associated with the HRQoL in people at risk of Pain/Discomfort and Anxiety/Depression are the quality-of-life dimensions with the most reported problems in the population at risk of T2D, largely among female participants and residents of Bogotá (a large city). The most frequent alterations to the quality-of-life dimensions in this type of population were the presence of some type of pain or discomfort, followed by anxiety or depression and problems in walking.

To our knowledge, there have been no studies establishing HRQoL or reporting the most common alterations in the populations at risk of future diabetes using the EQ-5D-3L in Colombia or South America. However, given that patients with prediabetes are at risk of progressing to the T2D and that many studies of patients with T2D used the EQ-5D questionnaire, we may draw some comparison with previous studies. Our data established that the dimension of quality of life with the greatest problem in patients at risk of developing T2D is Pain/Discomfort, which is in line with the current scientific evidence [15, 42, 43] revealing that patients with prediabetes (IFG) presented greater problems in the dimensions of physical functioning and body pain, similar to that observed by Tapp et al. in patients with IGT. Moreover, a prospective study using three different HRQoL questionnaires (SF-36, SF-6D, and 15D) reported that the deterioration of glycemic status negatively impacts the physical dimensions of HRQoL [43]. However, Seppälä et al. reported that prediabetes was not associated with HRQoL [16]. Among studies that used the EQ-5D questionnaire for populations with T2D, the results coincide with ours [44–46]. The dimension with maximum reports of moderate or severe problems in patients with T2D was Pain/Discomfort [44], consistent with the observations of Solli et al. [45] and Javanbakht et al. [46]. Recent studies of patients with diabetes confirmed that the most frequent alteration in the dimensions of quality of life was Pain/Discomfort [14, 47].

Several studies in patients with T2D have reported that after the pain/discomfort dimension, the Anxiety/Depression dimension was the second most reported problems [14, 45, 46]. However, in the population at risk of T2D, there is not enough scientific evidence to conclude that the presence of anxiety or depression is very prevalent among patients with prediabetes. However, it has been revealed that symptoms of depression were greater in women with prediabetes compared with participants with normal glucose metabolism [48]. A study in Greece using the 15D HRQoL questionnaire reported that patients with IGT presented problems in the dimension of “anguish” [19]. Finally, another study concluded that depression was more prevalent in people with T2D or at high risk of diabetes [49]. In our study, a large proportion of subjects at risk of T2D reported problems in the Mobility dimension, which is consistent with the results of Sakamaki et al. [44] and Makrilakis et al. [19].

The mean score on the visual analog scale (EQ-VAS) of the participants in our study was 74.3 ± 17.3. Unlike our study, previous research were all conducted in patients with T2D. For instance, a study of 1224 patients with diabetes, revealed a mean score of the EQ-VAS equivalent to 68 ± 18.0 [11], whereas in the study of Javanbakht et al., it was 56.8 (95% CI 56.15–57.5) [43]. In other recent studies of patients with T2D, the mean score on the same scale was 65.22 ± 9.32 [14] and 80 ± 12.9 [47], respectively. According to these results, it may be asserted that the perception of quality of life of patients at risk of future diabetes is better than those who suffer from the disease. In the present analysis, the female participants, despite presenting the same health state as the men, yielded smaller values on the EQ-VAS in the generic quality of life questionnaire. Also, women with T2D were found to have a poorer quality of life than men [11, 14, 44, 46]. Despite presenting the same complete health conditions as those from Bogotá, the participants from Barranquilla had lower values on the EQ-VAS. This agrees with the results of Rojas et al., who reported that citizens in the Atlantic region tended to express lower values compared to the other regions of Colombia [41]. Studies have shown that cities large countries present better living conditions due to economic development and the potentialization of the human condition that implies urban growth. However, several authors maintain, a city is large, promotes processes of spatial segregation and social exclusion, initiating the path to degradation of the quality of life of its inhabitants, since it is difficult to build a social fabric, a fundamental element of cooperation, solidarity and equity of a society important in the social determinants of the Health; evidencing that the geopolitical distribution of cities presents inequalities in all areas essentials of life that mark a diminished perception of the quality of life of the inhabitants [50, 51].

According to Javanbakht et al. [46], people living in larger cities are more likely to report problems in the self-care dimension. Moreover, place of residence was statistically significantly associated with the dimensions of Self-Care Usual Activities, and Anxiety/Depression in 300 patients with T2D in Birjand/Iran [14]. Sex was associated with the Pain/Discomfort and Anxiety/Depression dimensions, so women were more likely to report greater problems in these dimensions than men. This is agrees with findings in patients with T2D [11, 14, 46]. Alterations in the dimensions of Mobility and Usual Activities were more frequently reported by older participants (55 years or more) as in other studies as well [14, 46, 52]. These findings may be important for clinical practice because the use of HRQoL measures to evaluate the health, satisfaction and well-being results of the patient, provide an tool in the quality of patient care and in the decision-making process. Therefore, knowing the perception of quality of life (EQ-VAS), identifying factors related to HRQoL and having population scales are essential to characterize and interpret aspects of interest in persons at risk of diabetes [53, 54]. We encourage further inquiry into the HRQL of people at risk of diabetes, although we work with participants from lower socioeconomic strata, we recommend extending it to the rest of the socioeconomic strata.

Naturally, our study has some limitations. First, a set of values for the EQ-5D-3L in Colombia has not yet been established. Therefore, we were unable to report here the EQ-5D score or utility weights of the population at risk of diabetes (We hope to estimate the EQ-5D score using the set of values recommended for Colombia in future studies), and we were also unable to show the association or significant differences between the EQ-5D and EQ-VAS scores [9, 41]. The low sensitivity and ceiling effect of the EQ-5D-3L compared to the 5-level version (EQ-5D-5L) places another limitation on the responses of the participants [21–24]. However, we prefer to use the 3-level version because this study is part of the health economics research to investigate the cost effectiveness of diabetes prevention programs and the guidelines for economic evaluation in Colombia recommend using the system EQ-5D-3L [55]. Furthermore, all the participants in our study belong to the lowest socioeconomic level. Therefore, our results do not allow us to generalize populations with different socioeconomic levels or are not necessarily applicable to the general population. We also lacked information on potentially useful clinical variables such as comorbidities and current treatments of the patients, which could behave as a confounding factor or have effects on the HRQL found in this study. Finally, as it is a cross-sectional study, the observed associations are not necessarily causal.

## Conclusions

As sex, age, living place, impaired glucose tolerance, treatment for hypertension, and educational level were predictors of HRQoL in people at future risk of T2D, greater attention should be paid to these determinants in order to design, reorient and implement strategies that to achieve better health outcomes. Due to the characteristics related to this study, studies are required in populations of other socioeconomic strata that allow extrapolating the results to the entire population at risk of diabetes and identifying aspects of interest in this population. Nevertheless, future studies should focus on validating utility values in this type of population, estimating HRQoL according to glycemic status and determining if self-reported perceptions are consistent with the level of utility of the participants.

## Data Availability

The datasets presented in this article are not readily available because The PREDICOL Project is a Project that is currently in progress, therefore the database according to the regulations may not be published until the end of the study, for any requirement you can contact the authors. Requests to access the datasets should be directed to laanillo@uninorte.edu.co. All authors certify that they have no affiliations with or involvement in any organization or entity with any financial interest or non-financial interest in the subject matter or materials discussed in this manuscript.
